# Comprehensive analysis of clinicopathologic and prognostic features in locally advanced thyroid papillary cancer

**DOI:** 10.1016/j.bjorl.2024.101553

**Published:** 2025-01-03

**Authors:** Liang Jiwang, Ye Dongman, Fang Fengqin, Zhao Yuejiao

**Affiliations:** a Cancer Hospital of China Medical University, Liaoning Cancer Hospital & Institute, Department of Head and Neck Surgery, Liaoning Province, China; b Cancer Hospital of China Medical University, Liaoning Cancer Hospital & Institute, Department of Medical Imaging, Liaoning Province, China

**Keywords:** Locally advanced papillary thyroid cancer, Clinicopathological feature, Recurrence, Nomogram

## Abstract

**Objective:**

Differentiated thyroid cancers tend to excellent long-term survival after surgery. However, Locally Advanced Papillary Thyroid Cancers (LAPTCs) have poor prognosis. This study was to investigate the clinicopathologic features of LAPTC and the risk factors that affect its postoperative recurrence. We aimed to construct a nomogram to predict Recurrence-Free Survival (RFS) in LAPTC.

**Methods:**

We retrospectively reviewed the data of patients who underwent surgery from 2011 to 2020 at a single institution. Univariate and multivariate analyses were used to investigate the clinicopathologic features of LAPTC. The risk factors that affecting recurrence of LAPTC were screened. The risk factors were subsequently integrated to establish a predictive model. C-index, receiver operating characteristic curve and calibration curve were used to validate the accuracy. A Decision Analysis Curve (DCA) was used to evaluate the clinical value.

**Results:**

A total of 2530 Early Papillary Thyroid Cancer (EPTC) and 764 LAPTC were enrolled. Compared to EPTC, the proportion of male, bilaterality, multifocality, tumor size > 1 cm, ETE, central lymph node metastasis, and lateral lymph node metastasis was higher in LAPTC (all *p* < 0.05). Univariate and multivariate analyses found that age ≥ 55-years-old, male, and ETE were independent risk factors for the LAPTC patients’ RFS (all *p* < 0.05). C-index of the nomogram was 0.79. The AUC values of nomogram were 0.767 (95% CI 0.626‒0.909) and 0.798 (95% CI 0.669‒0.926) for 3- and 5-year RFS, respectively. The calibration curves of the nomogram showed good accuracy and consistency, and the DCA curves exhibited that the model had good clinical utility.

**Conclusion:**

Male patients who with tumors tumor > 1 cm, bilaterality, multifocality, and ETE are more likely to become LAPTC. LAPTC patients with age ≥ 55-years-old, tumor size > 1 cm, and ETE are more likely to have postoperative recurrence. The model can help surgeons to predict 3- and 5-year RFS in LAPTC.

**Evidence level:**

This article’s evidence level is four. Level 4 evidence, the case series, comes in the form of a group of patients subjected to surgical procedure. Authors may detect a statistically significant and clinically relevant outcome.

## Highlights

• There are no studies about the models for predicting recurrence in LAPTC.
• We established a nomogram for predicting 3- and 5-year RFS of LAPTC patients.
• The number of LAPTC patients is relatively larger than other study.

## Introduction

The incidence of thyroid cancer has been steadily increasing over the past few decades.^1^ According to the Global Cancer Observatory 2018 database, this disease has become the 9^th^ most common human malignancy, with approximately 570,000 new cases per year.^2^, ^3^ Several reasons for the epidemiological development have been under debate. It can be attributed to significant improvements of diagnostic techniques, especially higher sensitivity of ultrasound. Well-Differentiated Thyroid Cancer (WDTC), which includes Papillary Thyroid Cancer (PTC) and follicular cancer, constitutes approximately 80% of all thyroid malignancies. PTC is associated with a good prognosis, and a higher survival rate when diagnosed early with a 10-year survival rate of over 95%.^4^ According to the current guidelines, the treatment in most patients with Differentiated Thyroid Cancer (DTC) is surgery. With that approach, thyroidectomy eliminates any possibility of residual multifocal disease and allows the use of radioactive iodine for therapeutic purposes. However, around 13%‒15% of PTC patients still have locally aggressive and advanced tumors with worse prognosis.^5^, ^6^ PTC is defined as “locally advanced” in the presence of an extra thyroid extension, i.e., when the tumor passes thyroid capsule infiltrating the surrounding tissues.^7^ Many authors proposed this Extrathyroidal Extension (ETE) was one of the main risk factors for developing postoperative recurrence, and most patients who die from DTC had significant local complications from locally advanced disease.^8^

Currently, there are few relevant reports focused on Locally Advanced PTC (LAPTC). No define conclusion has been drawn from these studies. Thus, identification of clinicopathologic features is likely to facilitate optimal therapeutic decision in LAPTC. We retrospectively investigated the incidence rate and the related clinicopathologic characteristics of LAPTC. We then examined the risk factors that affecting postoperative relapses, attempting to reveal the potential independent predictors. We also established a nomogram to predict the postoperative recurrence in LAPTC patients, and these outcomes could assist greatly in decision-making regarding further treatment.

## Methods

### Patients

We retrospectively identified 3,782 patients who underwent thyroid surgical treatment in our hospital, from January 2011 to December 2020. The present study was accordance with the Declaration of Helsinki and was approved by ethical committees of our hospital (nº 20181207). The eligibility criteria were: (1) Patients without previous history of radiation and other head and neck malignant tumors; (2) PTC had been confirmed by histopathological examination; (3) The medical records of patients were complete. The patients who had non-PTC, and metastatic carcinoma from other organs were excluded from this study. Patients with previous thyroid surgery were excluded. After the inclusion and exclusion criteria, 3,294 PTC patients were included.

### Data collection

The following clinicopathological factors were evaluated and recorded: age, gender, bilaterality, multifocality, tumor size, Hashimoto Thyroiditis (HT), ETE, Lymph Node Metastasis (LNM), and TNM staging. Bilaterality was defined as PTC present in both thyroid lobes. Multifocality was defined as two or more tumors in one or both lobes. Any extension exceeding the thyroid gland was defined as ETE. Early PTC (EPTC) was defined as T_1-3a_N_0-1a_M_0_; LAPTC was defined as T_1-3a_N_1b_M_0_/T_3b-4_N_0-1_M_0_. All patients were classified according to the 8^th^ TNM staging system.^9^

### Surgery treatment

All surgical strategies have been performed according to the Chinese Thyroid Association clinical practice. The methods of primary lesion dissection used in our department included thyroid lobectomy with or without isthmectomy and total thyroidectomy. The methods of lymph node dissection included central compartment node dissection and lateral neck dissection. The thyroid hormone suppressive therapy was performed to all patients after surgery.

### Follow-up

All patients were examined at 3-, 6- and 12-months after the initial treatment and yearly thereafter, or more frequently according to the clinical course. Patient progress was followed by physical examination, ultrasound and CT to identify local thyroid remnant, lymph node and distant metastasis. Local recurrence refers to the recurrence of the ipsilateral lobe, bronchial stump, or local lymph nodes. Distal metastasis refers to the recurrence and metastasis of distal organs, such as the lung, liver, brain, and bone. Distant metastasis was defined when both local recurrence and distant metastasis occur. Recurrence-Free Survival (RFS) was defined as the time interval from the day of surgery to the last day of follow-up when the tumor recurred and metastasized.

### Statistical analysis

Univariate analysis was performed using Pearson’s Chi-Square test and Fisher’s exact test as appropriate. Statistically significant results obtained from univariate analysis were submitted to multivariate logistic regression. RFS was analyzed using Kaplan-Meier survival curve, and comparison was made using the log-rank test. A nomogram was formulated based on the results of cox regression analysis. The discriminative ability of the predictive nomogram was assessed by Harrell’s Concordance Index (C-index). The specificity and sensitivity of the nomogram were assessed via the Receiver Operating Characteristics (ROC) curve. The calibration curve was used to evaluate the predictive performance by comparing the nomogram-predicted and actual observed 3- and 5-year RFS. The Decision Curve Analysis (DCA) was generated to evaluate the clinical usefulness of the nomogram by quantifying the net benefits at different threshold probabilities. A *p* < 0.05 represented a statistically significant difference. All statistical analyses were performed using the SPSS 22.0 statistical package (SPSS, Inc., Chicago, IL, USA), and R language software and the rms package.

## Results

### Patient characteristics

Of the 3,294 patients, there were 688 males and 2,606 females. The mean age was 44.7 ± 37.5-years-old (range from 10 to 85). Bilaterality was found in 875 patients, multifocality in 1032 patients, and ETE in 443 patients. The mean tumor size was 1.35 ± 0.93 cm, and the rate of papillary thyroid microcarcinoma was 49.3%. PTC coexistent with HT was observed in 889 patients. Central Lymph Node Metastases (CLNM) were identified in 1,331 patients, and Lateral Lymph Node Metastases (LLNM) in 494 patients. There were 3,052 patients with stage I disease, 176 with stage II, and 66 with stage III. Among these patients, 2,530 patients were EPTC, and 764 patients were LAPTC. We found gender, bilaterality, multifocality, tumor size, HT, ETE, CLNM, and LLNM showed statistical differences between EPTC and LAPTC groups (*p* < 0.01, Table 1).

**Table 1:** Clinicopathologic features of patients with EPTC and LAPTC.

	EPTC	LAPTC	p
	(n = 2530)	(n = 764)	
Age (years)			0.800
≥ 55	526	162	
< 55	2004	602	
Gender			0.000
Male	476	212	
Female	2054	552	
Bilaterality			0.000
Present	548	327	
Absent	1982	437	
Multifocality			0.000
Absent	1866	396	
Unilateral multifocal PTC	121	45	
Bilateral multifocal PTC	543	323	
Tumor size (cm)			0.000
≤ 1	1469	156	
> 1	1061	608	
Hashimoto thyroiditis			0.000
Present	725	164	
Absent	1805	600	
ETE			0.000
Absent	2530	321	
Strap muscle (+)	0	224	
Surrounding organ (+)	0	162	
Strap muscle and surrounding organ (+)	0	57	
CLNM			0.000
Present	817	514	
Absent	1713	250	
LLNM			0.000
Present	0	494	
Absent	2530	270	

### Follow-up analysis

Analysis of the follow-up data of the study population showed that the median follow-up time was 2,079 days (range 1,097‒4,737 days). Postoperative recurrence intervals from initial operation ranged from 69 days to 2,858 days. By the end of follow-up, 18 LAPTC patients had evidence of metastasis or recurrence. In these patients, ages ranged from 28 years to 69 years old. Bilaterality was detected in 13 patients, multifocality was found in 13 patients, and ETE was observed in 15 patients. Only one patient exhibited HT. CLNM and LLNM were identified in 13 and 11 patients, respectively. Three patients were found recurrence within one year, 14 patients developed relapse within two to five years, and one patient showed recurrence over five years later. Recurrence occurred once in 17 patients, twice in only one patient. The sites of recurrence or metastasis were lymph node, mediastinal metastasis, larynx, tracheal stoma edge, diaphragm, lung and bone.

### Univariate and multivariate analysis of recurrence in LAPTC

We found that age (*p* = 0.000), gender (*p* = 0.003), bilaterality (*p* = 0.014), multifocality (0.029), and ETE (*p* = 0.004) were significantly associated with recurrence in LAPTC patients. Multivariate analysis revealed that age (*p* = 0.000), gender (*p* = 0.001), and ETE (*p* = 0.048) were independent risk factors (Table 2).

**Table 2:** Univariate and multivariate analysis of risk factors of recurrence in LAPTC patients.

	Recurrence (n = 18)	Non-recurrence (n = 746)	Univariate analysis	Multivariate analysis		
			p	OR	95% CI	p
Age (years)			0.000	0.143	0.050‒0.405	0.000
≥ 55	11	151				
< 55	7	595				
Gender			0.003	0.181	0.065‒0.501	0.001
Male	11	201				
Female	7	545				
Bilaterality			0.014	7.792	0.185‒327.332	0.282
Present	13	314				
Absent	5	432				
Multifocality			0.029	1.356	0.205‒8.963	0.752
Absent	5	391				
Unilateral multifocal PTC	0	45				
Bilateral multifocal PTC	13	310				
Tumor size (cm)			1.000			
≤ 1	3	153				
> 1	15	593				
Hashimoto thyroiditis			0.143			
Present	1	163				
Absent	17	583				
ETE			0.004	0.620	0.386‒0.996	0.048
Absent	3	318				
Strap muscle (+)	6	218				
Surrounding organ (+)	4	158				
Strap muscle and surrounding organ (+)	5	52				
CLNM			0.802			
Present	13	501				
Absent	5	245				
LLNM			0.804			
Present	11	485				
Absent	7	261				

### Selection of prognostic factors and establishment of nomogram

**Fig. 1:**
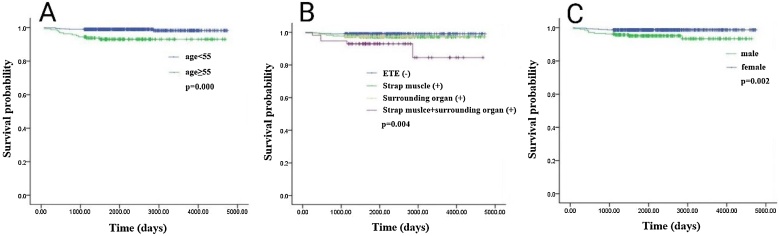
Kaplan-Meier curves estimating RFS. (A) Age; (B) ETE; (C) Gender.

**Fig. 2:**
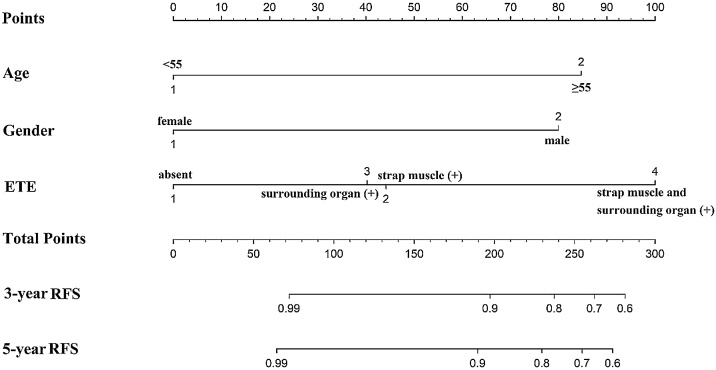
The nomogram for predicting 3- and 5-year RFS in LAPTC patients.

We selected the above three factors (age, gender, and ETE) to analyze the influence on the patients’ RFS. As shown in Fig. 1A, the RFS time was significantly shorter in patients with age ≥ 55-years-old than in younger individuals (mean time 73.2 vs. 77.0 months, *p* = 0.000). Kaplan-Meier curves revealed a significantly shorter RFS time for patients with ETE (mean time 72.4 vs. 76.5 months, *p* = 0.004, Fig. 1B). The RFS time was also significantly shorter in male gender patients than female patients (mean time 74.4 vs. 76.1 months, *p* = 0.002, Fig. 1C). The prognostic factors (age, gender, and ETE) were included to build a nomogram model for predicting 3- and 5-year RFS in LAPTC patients (Fig. 2).

## Nomogram validation and evaluation

**Fig. 3:**
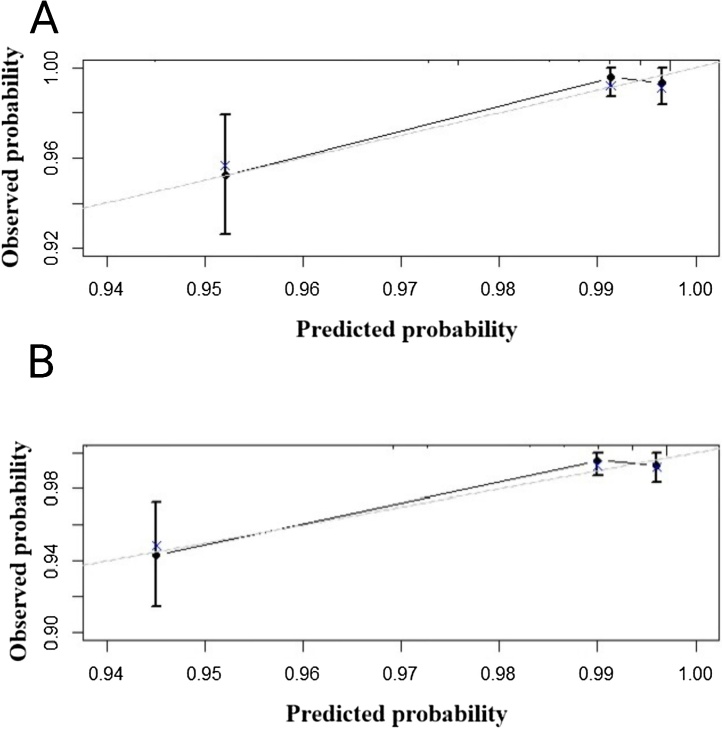
Calibration plots for RFS. (A) 3-year RFS; (B) 5-year RFS.

**Fig. 4:**
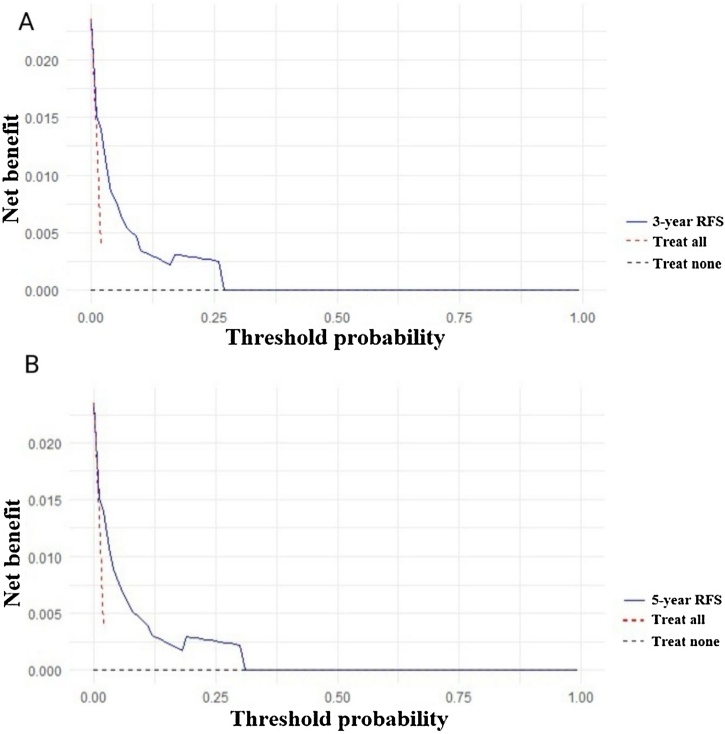
DCA of the nomogram for RFS. (A) 3-year RFS; (B) 5-year RFS.

**Fig. 5:**
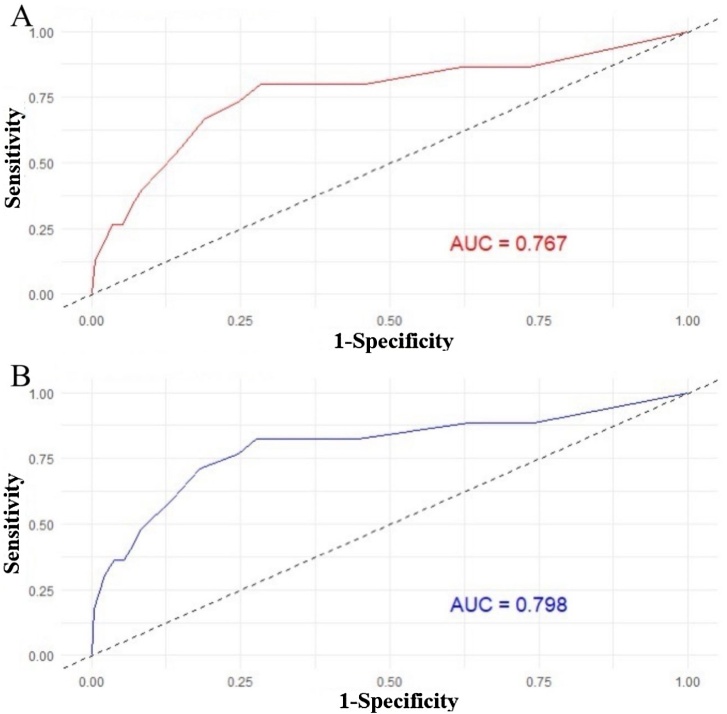
The receiver operating characteristic curve for RFS prediction model. (A) 3-year RFS; (B) 5-year RFS.

To test the prediction value of the nomogram model, the C-index was further calculated to be 0.79 (95% CI 0.66‒0.92), and it indicated that this model had a good discrimination ability for predicting the postoperative RFS. The calibration curves for RFS at 3- and 5-year showed good agreement between the predicted and actual values of the nomogram (Fig. 3A‒B). The DCA results suggested that this nomogram had excellent clinical applicability (Fig. 4A‒B). As shown in Fig. 5A‒B, the ROC curves of the nomogram showed good discriminatory ability, with AUC values of 0.767 (95% CI 0.626‒0.909) and 0.798 (95% CI 0.669‒0.926) for 3- and 5-year RFS, respectively. The sensitivity of 3- and 5-year RFS was 80.0% and 83.0%, respectively. The specificity of 3- and 5-year RFS was 72.0% and 72.0%, respectively.

## Discussion

Over the last several decades, the incidence of thyroid cancer has significantly increased, especially since papillary thyroid microcarcinomas are more frequently diagnosed. It exhibits prognostically favorable compared to other cancers, with disease-specific death rates as low as 1.3%.^10^ This can be explained as improved pre-operative screening and more well-defined surgical management of DTC. WDTC comprises majority of PTCs, with 10%‒15% presenting as locally advanced diseases. It is noteworthy that about 15% of patients need salvage treatment because of the uncontrolled locoregional recurrence and distant metastasis.^11^, ^12^ According to the American Thyroid Association and Japanese Society of Thyroid Surgery high-risk disease guidelines, surgery is the primary treatment. However, the optimal surgical approach for recurrence of LAPTC is still controversial.^13^, ^14^ Because LAPTC can involve the adjacent tissues or organs, the challenge for these cancers is to be ideally radical, weighting a potential significant morbidity.^15^ Thus, identification of the risk factors associated with recurrence is important to avoid underestimation and inadequate treatment for those patients with LAPTC.

The rate of LAPTC was 23.2% in our hospital that spans 10-years. In comparison to EPTCs, LAPTCs were characterized by more frequent male gender, bilaterality, multifocality, larger tumor, presence of ETE and CLNM. Although most of PTC are detected in an early phase and are small size at present, there has been an increasing number of larger cancers that manifest with locally advanced disease, mostly defined by ETE into adjacent soft tissue, or even worse into nearby organs. In this study, the rate of ETE in LAPTC patients was 58%, and the rate of ETE in LAPTC patients who had postoperative recurrence was 83.3%. After initial treatment, if PTC patient has gross disease, local recurrence is more frequent, especially for those who present with T4a tumors involving recurrent laryngeal nerve, trachea, esophagus, or bulky metastatic nodes. As having an important effect on different stage-related survival rate, particular significance has recently been attributed to ETE in the new 8^th^ edition AJCC Cancer Staging Manual.^8^ Thus, we need to pay more attention to this risk factor, because it can predict a worse prognosis.

As is known to us, PTC can spread via lymphatic ducts, which results in recurrence, metastases, and even death.^16^, ^17^ Many factors can affect PTC recurrence, and the conclusion has not yet been reached. In this study, the rate of recurrence in LAPTC was 2.4%. Univariate analysis indicated that factors leading to a higher likelihood of recurrence include increasing age, male gender, bilaterality, multifocality, and ETE. Through multivariate analysis, we found age ≥ 55-years-old, male, and ETE were independent risk factors for RFS of LAPTC. At present, there is still controversy regarding multifocality and bilaterality in PTC and survival outcomes. Some authors described that multifocal PTC patient had higher recurrence rate compared to solitary PTC.^18^ Kim et al. in their study found that the number of multifocal tumors rather than the location was significant predictive factor for RFS.^19^ Qu et al. proposed that bilaterality rather than unilateral multifocality was an independent risk factor for locoregional recurrence, distant metastasis, and cancer death in PTC.^20^ However, although we found the association of bilaterality and multifocality with increased risk of recurrence, they were not the independent risk factors by multivariate analysis. Moreover, our result revealed that the rate of male is 61.1%, and recurrence was associated with increasing age, especially in patients ≥ 55-years-old, which was similar to previous results.^17^, ^21^, ^22^ We speculated the explanation which might be the normally higher basal metabolic rate in male patients. This can incite an overactive proliferation of tumor cell and lead to more metastasis. Simultaneously, age ≥ 55-years-old was a risk factor for gross ETE, and it was possibly due to a longer period of tumor growth in older patient.

Currently, for LAPTC patients with or without recurrence, comprehensive assessments based on pathological diagnosis and medical imaging are important for medical decision-making and follow-up. In our hospital, the different therapeutic choices have been tailored to the characteristics of PTC patients, and the surgical approach has led to encouraging result during the follow-up in this study: 99.7% of the LAPTC patients with available follow-up are currently alive. The 3- and 5-year RFS was 98.0% and 97.8%, respectively. Only 18 LAPTC patients developed relapse, and most of recurrence were in regional lymph node metastases. They were subsequently successfully treated by re-surgery and adjuvant therapy. Through analysis of clinical and follow-up data of LAPTC patients undergoing surgery, we then explored the prognostic factors of postoperative RFS in these patients and tried to establish a prognostic evaluation system suitable for thyroid cancer patients. We attempted to construct a nomogram model based on age, gender, ETE to predict RFS of LAPTC patients. The nomogram demonstrated potential value in clinical practice. To our knowledge, this study established the first nomogram model for predicting postoperative RFS in LAPTC. It allowed clinicians to comprehensively assess the risk of recurrence and develop treatment plans appropriated for individual patients.

There were still some limitations. Firstly, this study was conducted in a single center and was retrospective, which may result in certain selection bias. Retrospective studies rely on existing medical records, completeness and the accuracy of which can be affected by various factors. It may lead to some important variables being excluded from the analysis, potentially impacting the accuracy of our predictive model. Secondly, this study only conducted the internal validation of the nomogram and lacked external validation, which may have some drawbacks, such as alteration of data distribution, issues with result stability, and potential risk of overfitting. Thus, it is imperative to prioritize additional external validation cohorts from further studies to comprehensively assess the viability of the nomogram in the present study. Thirdly, the nomogram of our study might have lower predictive ability in other areas due to factors such as race and different etiology constituting distribution in different regions. Further analysis and evaluation should be carried out in multicenter large-sample clinical data to validate the model, and enhance its reliability and applicability.

In conclusion, although LAPTCs are uncommon, when encountered, they are challenging to manage especially when these patients had relapse. Our results indicated that LAPTC was associated with an aggressive clinical course and should therefore accordingly be assertively managed. Several risk factors including older age, male gender, and ETE were significantly associated with higher recurrence risk and shorter RFS for LAPTC patients. We established a prognostic model, and it showed reliable performance and contribute to the individualized treatment. Further research is required to identify recurrence rate and the associated risk factors, which could improve the early detection, and deliver better treatment and surveillance.

## Funding

This work was supported by the Natural Science Foundation of Liaoning Province (2023-MS-059, nº 20180530038).

## Declaration of competing interest

We do not have any financial relationship that may lead to a conflict of interest in relation to the submitted manuscript.
